# CHA_2_DS_2_‐VASc and PESI scores are associated with right ventricular dysfunction on computed tomography pulmonary angiography in patients with acute pulmonary thromboembolism

**DOI:** 10.1002/clc.23786

**Published:** 2022-02-07

**Authors:** Toktam Alirezaei, Zahra Mahboubi‐Fooladi, Rana Irilouzadian, Ali Saberi Shahrbabaki, Haniyeh Golestani

**Affiliations:** ^1^ Clinical Research Development Unit, Shohada‐e‐Tajrish Hospital Shahid Beheshti University of Medical Sciences Tehran Iran; ^2^ Department of Radiology Shahid Beheshti University of Medical Sciences Tehran Iran; ^3^ School of Medicine Shahid Beheshti University of Medical Sciences Tehran Iran

**Keywords:** CHA_2_DS_2_‐VASc score, computed tomography pulmonary angiography, pulmonary embolism, pulmonary embolism severity index, right ventricular dysfunction, risk stratification

## Abstract

**Background:**

Accurate risk stratification is the most important step in the management of patients with acute pulmonary thromboembolism (PTE). Pulmonary embolism severity index (PESI) is a clinical tool for PTE risk stratification. CHA_2_DS_2_‐VASc score, a risk assessment tool in patients with atrial fibrillation, is recently considered for acute PTE. The presence of right ventricular (RV) dysfunction in imaging is more efficient in acute PTE risk evaluation.

**Hypothesis:**

This study aims to evaluate the association between CHA_2_DS_2_‐VASc and PESI score and each of them with RV dysfunction on computed tomography pulmonary angiography (CTPA).

**Methods:**

One hundred eighteen patients with a definite diagnosis of PTE were entered. The CHA_2_DS_2_‐VASc and PESI scores were calculated for all of them. RV dysfunction including an increase in RV to left ventricular diameter ratio, interventricular septal bowing, and reflux of contrast medium into the inferior vena cava was examined by CTPA.

**Results:**

PESI and CHA_2_DS_2_‐VASc scores were significantly associated with RV dysfunction. In addition, different classes of PESI scores were correlated with RV dysfunction. Moreover, this study showed that the CHA_2_DS_2_‐VASc score and PESI score had a positive correlation. The area under the curve value for the CHA_2_DS_2_‐VASc score was 0.625 with 61.54% sensitivity and 60.0% specificity for predicting RV dysfunction while for PESI score was 0.635 with 66.7% sensitivity and 60.0% specificity.

**Conclusion:**

This study showed that not only CHA_2_DS_2_‐VASc and PESI scores are positively correlated, but they are both associated with RV dysfunction diagnosed by CTPA. CHA_2_DS_2_‐VASc and PESI scores are able to predict RV dysfunction.

## 1 INTRODUCTION

Acute pulmonary thromboembolism (PTE) is a life‐threatening emergency that is responsible for nearly 200 000 deaths each year alone.^1^ Acute PTE manifests with a broad spectrum of presentations from mild clinical features to severe life‐threatening conditions and even death. The proper diagnostic approach and accurate risk stratification are essential for decreasing the morbidity and mortality rates of PTE.^2^ In addition to making the diagnosis, the physician must decide the settings the patient should be managed and the best treatment that should be initiated.^3^ Clinical evaluation and assessment of right ventricular (RV) size and function are two key components for risk stratification.^4^ Computerized tomography pulmonary angiography (CTPA) is the choice method in the diagnosis of PTE with excellent sensitivity and specificity. Moreover, CTPA helps with evaluating the prognosis of patients with PTE. CTPA shows the four‐chamber view of the heart and demonstrates RV dysfunction as an increase in RV to left ventricular (RV/LV) diameter ratio, interventricular septal bowing, and reflux of contrast medium into the inferior vena cava (IVC).^5^, ^6^ The European Society of Cardiology (ESC) guidelines recommend clinical, imaging, and laboratory evaluation of PTE severity, mainly related to the presence of RV dysfunction, to risk‐stratify a patient with acute PTE more thoroughly.^7^ The presence of RV dysfunction in imaging studies seems to act as an independent variable for predicting high mortality in acute PTE.^8^, ^9^ A recent study suggests that in the setting of acute PTE, which requires an accurate risk stratification tool, the presence of RV dysfunction alone is sufficient and superior for this purpose.^10^ The severity of pulmonary embolism is categorized as high, intermediate‐high, intermediate‐low, and low risk. High‐risk PTE is defined by signs of overt RV failure and hemodynamic instability. In hemodynamically stable patients, acute RV dysfunction with accompanying elevated cardiac biomarker levels is presented in the intermediate‐high‐risk group. Patients with intermediate‐low risk PTE present with normal RV function and/or normal cardiac biomarker levels. The remaining group of patients is classified as low risk. This classification in patients with acute PTE is a guide for choosing the treatment method. Anticoagulation should be initiated in patients with high or intermediate risk of PTE during the time that diagnostic workup is advancing. While thrombolysis is the choice in patients with a high risk of acute PTE or intermediate‐risk patients on anticoagulation with hemodynamic deterioration, early discharge, and continuation of treatment at home is suggested for carefully selected patients with low risk of acute PTE.^7^

The pulmonary embolism severity index (PESI) specifies 11 features from history, demographics, and clinical findings and is a clinical risk stratification tool. Some studies suggest the association between the presence of RV dysfunction and PESI in patients diagnosed with PTE.^11^, ^12^ The CHA_2_DS_2_‐VASc (C: congestive heart failure, H: hypertension, A: age of ≥75 years, D: diabetes mellitus, S: previous stroke, V: vascular disease, A: age between 65 and 74 years, Sc: female gender) has been shown as a useful clinical score to evaluate the thromboembolism risk and to administer the anticoagulant in patients diagnosed with nonvalvular atrial fibrillation.^13^ The components of the CHA_2_DS_2_‐VASc score are simple and practical. The CHA_2_DS_2_‐VASc score has been considered for predicting the prognosis and mortality of coronary artery bypass grafting surgery and various cardiovascular diseases such as acute myocardial infarction and heart failure.^11^, ^13^, ^14^, ^15^, ^16^, ^17^, ^18^ It has been reported that a higher CHA_2_DS_2_‐VASc score is associated with cardiovascular events such as ischemic stroke, thromboembolism, and death in patients without atrial fibrillation.^19^, ^20^, ^21^ In a study in acute PTE patients with cardiac sinus rhythm, the CHA_2_DS_2_‐VASc score has been suggested to provide further prognostic information besides the PESI score.^22^ A recent study showed the association between CHA_2_DS_2_‐VASc score and echocardiographic indices of RV dysfunction in patients with acute PTE.^23^ In this study, we investigated the association of the CHA_2_DS_2_‐VASc score with the PESI score, association and predictive values of PESI score and CHA_2_DS_2_‐VASc for RV dysfunction on CTPA and assessed the correlation between different variables of these two scores and RV dysfunction.

## 2 METHODS

This was a retrospective study that was conducted on patients over 18 years of age with a diagnosis of acute PTE by CTPA during the period between January 2019 and December 2020. Exclusion criteria were a history of previous thromboembolism, use of thrombolytic and anticoagulant agents, a history of the previous diagnosis of RV dysfunction, the presence of intracardiac lesions, and any concomitant disorder affecting the hemodynamics of the patient (e.g., septic shock). As a result, 118 eligible patients were included in the study consecutively after signing the written informed consent. The study was approved by the ethics committee of the Shahid Beheshti University of Medical Sciences (Ethics code: IR.SBMU.RETECH.REC.1400.179).

All CT scan images were acquired using a 16‐section multidetector CT scanner based on the standard CTPA protocol for PTE and 20 s after injection of 100 ml contrast media at a rate of 5 ml/s. Careful evaluation of the CTPA images was made by an expert radiologist to detect signs of RV dysfunction by analyzing RV/LV diameter ratio, interventricular septal bowing and reflux of contrast medium into the IVC. LV and RV dimensions were measured exactly below the atrioventricular leaflets and defined as the distance between the endocardium of the septum with the ventricular wall. Reflux of contrast medium was considered noticeable only when the reflux was seen into the IVC and hepatic veins. Diagnosis of RV dysfunction was based on the presence of at least one of these three criteria: (1) RV/LV diameter ratio ≥0.9; (2) bowing of interventricular septum; and (3) reflux of contrast medium into the IVC.^24^

A clinician reviewed charts and scored PESI and CHA_2_DS_2_‐VASc, blinded to patient outcomes. PESI score includes 11 features and is calculated by summing the patient's age in years (age > 80 years) and point assigned for each of the 10 variables as follows: 10 points for the male gender, 30 points for a history of malignancy, 10 points for a history of heart failure, 10 points for a history of chronic lung disease, 20 points for the presence of tachycardia (heart rate >109 beats/min), 30 points for systolic hypotension (systolic blood pressure <100 mmHg), 20 points for the presence of tachypnea (respiratory rate ≥30 breaths/min), 20 points for a temperature less than 36°C, 60 points for an altered mental status, and 20 points for arterial oxygen saturation less than 90%. According to the PESI score, patients were categorized into five classes including Class I (<65 points: very low risk), Class II (66–85 points: low risk), Class III (86–105 points: intermediate risk), Class IV (106–125 points: high risk), and Class V (>125 points: very high risk).

The CHA_2_DS_2_‐VASc score was calculated for each patient as follows: 1 point for each of congestive heart failure, systemic hypertension, age between 65 and 74 years, diabetes mellitus, vascular disease, and female gender and 2 points for each of age equal or more than 75 years and history of the previous stroke, transient ischemic attack, or thromboembolism.

All statistical analyses were performed using the SPSS software, version 23 (SPSS Inc.). The mean ± *SD* or quartiles were used for the analyses of parametric variables. Frequency and percentage were used to represent categorical variables. Kolmogorov–Smirnov statistics was used for checking the normal distribution assumption of continuous variables. In addition, simple linear logistic and logistic regression were used to perform the analyses. Independent samples *t*‐test was used for variables with normal distribution and Mann–Whitney *U* test was used to assess in absence of normal distribution. Odds ratio (OR) was reported for the effective variables. To evaluate CHA_2_DS_2_‐VASc and PESI scores accuracy at predicting RV dysfunction, we used the receiver operating curve (ROC) analysis by MedCalc. ROC curve data were reported as the area under the curve (AUC). *p* < .05 was considered as the statistical significance level.

## 3 RESULTS

In this study, 118 patients including 60 (50.8%) males and 58 (49.2%) females were entered. The patients aged from 18 to 93 years old with a mean ± *SD* of 59.64 ± 20.6 years. The calculated PESI score was ranged from 27 to 283 with a mean ± SD of 106.5 ± 46.9. The prevalence of PESI score risk factors among patients showed that male gender, arterial O_2_ saturation of less than 90%, and heart rate more than 109 beats/min were the most prevalent risk factors (Table 1). The CHA_2_DS_2_‐VASc score among patients ranged from 0 to 8 with a mean of 2.30 ± 2.08. The prevalence of CHA_2_DS_2_‐VASc score risk factors among patients demonstrated female gender, hypertension, and age ≥75 years old as the most common risk factors (Table 1).

**Table 1:** Distribution of PESI and CHA_2_DS_2_‐VASc score risk factors among the patients

Variable	Patients = 118 (%)
PESI risk factors	
Male	60 (50.8%)
Arterial O_2_ saturation <90%	51 (43.2%)
Heart rate >109 beats/min	30 (25.4%)
History of heart failure	28 (23.7%)
History of malignancy	28 (23.7%)
Systolic blood pressure ≤100 mmHg	23 (19.5%)
Respiratory rate ≥30 breaths/min	19 (16.1%)
Altered mental status	16 (13.6%)
History of chronic lung disease	15 (12.7%)
Body temperature <36°C	1 (0.8%)
CHA_2_DS_2_‐VASc risk factors	
Female	58 (49.2%)
Hypertension	40 (33.9%)
Age ≥75	31 (26.3%)
History of congestive heart failure	26 (22%)
65 < age < 74	24 (20.3%)
Vascular diseases	22 (18.6%)
Diabetes mellitus	21 (17.8%)
TIA/TE/stroke	11 (9.3%)

Abbreviations: PESI, pulmonary embolism severity index; TE, thromboembolism; TIA, transient ischemic attack

Among 118 studied patients, 78 (66.1%) had RV dysfunction and 40 (33.9%) did not have RV dysfunction. Among the patients with RV dysfunction, 57 cases (48.3%) were in terms of reflux of contrast medium into the IVC, 56 cases (47.5%) were in terms of RV/LV diameter ratio ≥0.9 and 28 cases (23.7%) were in terms of interventricular septal bowing (Table 2). In patients with RV dysfunction, 37 (47.4%) cases had only one criterion, 19 (24.4%) had two criteria, and 22 (28.2%) had three criteria of RV dysfunction. The mean of PESI score and CHA_2_DS_2_‐VASc score in groups with and without RV dysfunction was evaluated and showed that both scores were significantly higher in patients with RV dysfunction. There was a statistically significant association between RV dysfunction and PESI score and also, CHA_2_DS_2_‐VASc score (*p* = .023 and041, respectively). Among RV dysfunction criteria, PESI score and CHA_2_DS_2_‐VASc score were correlated with reflux of contrast medium into the IVC (*p* = .010 and <.0001, respectively) (Table 2).

**Table 2:** Distribution of RV dysfunction and its three criteria among patients and their association with the mean of PESI score and CHA_2_DS_2_‐VASc score

	*N* (%)	PESI score, mean (*SD*)	*p* Value	CHA_2_DS_2_‐VASc score, mean (*SD*)	*p* Value
RV dysfunction					
Yes	78 (66.1%)	111.85 (42.98)	.023	2.58 (2.09)	.041
No	40 (33.9%)	91.98 (47.18)		1.75 (1.98)	
RV/LV diameter ratio ≥0.9					
Yes	56 (47.5%)	106.5 (38.65)	.753	2.01 (2.36)	.766
No	62 (52.5%)	103.85 (50.75)		2.16 (2.24)	
Intraventricular septum bowing					
Yes	28 (23.7%)	104.36 (31.4)	.920	2.36 (1.98)	.861
No	90 (76.3%)	105.34 (48.9)		2.12 (2.28)	
Reflux of contrast medium into IVC					
Yes	57 (48.3%)	116.11 (43.7)	.010	2.98 (2.12)	<.0001
No	61 (51.7%)	94.84 (44.57)		1.66 (1.84)	

Abbreviations: IVC, inferior vena cava; LV, left ventricle; PESI, pulmonary embolism severity index; RV, right ventricle.

The distribution of PESI score among patients with classification into five classes is shown in Table 3. Most of the patients were in Class V (30.5%) and the least was in Class II (11.9%). Different classes of PESI score were associated with RV dysfunction, meaning that patients without RV dysfunction are seen in Class I more than patients with RV dysfunction, and patients with RV dysfunction are seen in higher classes of PESI score (Table 3).

**Table 3:** Classification of PESI score among patients and their association with RV dysfunction

PESI score	*N* (%)	RV dysfunction		*p* Value
		Yes, *N* (%)	No, *N* (%)	
Class I (<66)	25 (21.2%)	10 (40%)	15 (60%)	.029
Class II (66–85)	14 (11.9%)	11 (78.6%)	3 (21.4%)	
Class III (86–105)	24 (20.3%)	15 (62.5%)	9 (37.5%)	
Class IV (106–125)	19 (16.1%)	15 (78.9%)	4 (21.1%)	
Class V (>125)	36 (30.5%)	27 (75%)	9 (25%)	

Abbreviations: PESI, pulmonary embolism severity index; RV, right ventricle.

The analysis also showed that among the risk factors of PTE, male gender and heart rate more than 109 beats/min were significantly correlated with RV dysfunction (Table 4). Moreover, the correlation between the CHA_2_DS_2_‐VASc score and the PESI score was measured and demonstrated a positive correlation with *r* = .523 (*p* < .0001). Results of the ROC analysis showed CHA_2_DS_2_‐VASc score had the AUC value of 0.625 (95% confidence interval [CI]: 0.3531, 0.712) for predicting RV dysfunction with 61.54% sensitivity and 60.0% specificity (*p* = .020). The AUC value of PESI score for predicting RV dysfunction was found to be 0.635 (95% CI: 0.542, 0.712) with sensitivity of 66.7% and specificity of 60.0% (*p* = 0.018) (Table 5 and Figure S1).

**Table 4:** Correlation of variables with RV dysfunction

Variable	beta	OR	95% CI	*p* Value
Age	0.252	1.287	0.599–2.765	.519
Male gender	0.023	1.023	1.004–1.043	.018
History of malignancy	−0.308	0.735	0.305–1.768	.491
History of heart failure	0.800	2.226	0.820–6.041	.116
History of chronic lung disease	−0.302	0.739	0.243–2.246	.594
Heart rate > 109 beats/min	1.194	3.302	1.155–9.443	.026
Systolic blood pressure ≤ 100 mmHg	0.742	2.100	0.717–6.153	.176
Respiratory rate ≥ 30 breaths/min	1.158	3.183	0.869–11.662	.081
Altered mental status	0.139	1.149	0.370–3.570	.810
Arterial O_2_ saturation < 90%	0.200	1.221	0.563–2.648	.613
Hypertension	0.445	1.56	0.679–3.586	.295
Age ≥ 75	0.74	1.095	0.831–5.400	.126
65 < age < 74	0.813	2.254	0.773–6.573	.137
Vascular diseases	0.38	1.462	0.524–4.085	.468
Diabetes mellitus	0.031	1.031	0.379–2.803	.952
TIA/TE/stroke	1.747	5.735	0.707–46.508	.102

Abbreviations: CI, confidence interval; OR, odds ratio; RV, right ventricle; TE, thromboembolism; TIA, transient ischemic attack.

**Table 5:** Results of ROC analysis for PESI score and CHA_2_DS_2_‐VASc score for prediction of RV dysfunction

	AUC (95% CI)	Specificity	Sensitivity	*p* Value
PESI score	0.635 (0.542–0.712)	60.0	66.7	.018
CHA_2_DS_2_‐VASc score	0.625 (0.531–0.712)	60.0	61.54	.020

Abbreviations: AUC, area under the curve; CI, confidence interval; PESI, pulmonary embolism severity index; ROC, receiver operating curve; RV, right ventricle.

## 4 DISCUSSION

This study aimed to demonstrate that CHA_2_DS_2_‐VASc and PESI scores are correlated with RV dysfunction. Onuk et al. concluded that the CHA_2_DS_2_‐VASc score can be used as a risk stratification and prognostic tool in the context of acute PTE, besides the PESI score.^22^ Yilmaz et al. reported that CHA_2_DS_2_‐VASc score can predict prognosis and management of acute PTE patients.^25^ Furthermore, a study by Gok et al. revealed CHA_2_DS_2_‐VASc score as an independent predictor of the RV dysfunction in patients with acute PTE with an AUC of 0.621, 70% sensitivity and 50% specificity.^11^ Similarly, our study showed that both scores are significantly higher in patients with RV dysfunction and the CHA_2_DS_2_‐VASc score had the AUC of 0.625 for predicting RV dysfunction with 61.54% sensitivity and 60.0% specificity. Although Gok et al. assessed RV dysfunction using echocardiography this study aimed to assess RV dysfunction by CTPA.

We also analyzed the predictive value of the PESI score for RV dysfunction which demonstrated an AUC of 0.635 with a sensitivity of 66.7% and specificity of 60.0%. Our study showed that higher classes of PESI score were associated with RV dysfunction, meaning that patients without RV dysfunction are seen in Class I more than patients with RV dysfunction, and patients with RV dysfunction are seen in higher classes of PESI score. Cho et al. conducted a study on 229 patients with acute PTE and divided the patients into two groups based on RV/LV diameter ratio. RV/LV diameter ratio >1 group showed significantly more patients with simplified PESI (sPESI) score of 2 and 3 in contrast to the RV/LV diameter ratio <1 group and score of 4 and 5 was only seen in the group with RV/LV diameter ratio >1^26^ which is consistent with the results of our study. In another study by Sanchez et al., the PESI score Class III, IV, and V were associated with moderate prognostic sensitivity (72%) and high negative predictive value (98%) for 30‐day adverse events.^27^ On the contrary, Soares et al. were unable to show any association between five classes of PESI score and RV/LV diameter ratio.^28^ Furthermore, a study in 779 PTE patients with sPESI of 0 showed that the RV/LV ratio ≥1.0 was not associated with an increase in all‐cause mortality.^29^

Different studies showed that the presence of RV dysfunction alone in the context of acute PTE is sufficient for the purposes of risk stratification and adverse outcome.^10^, ^30^ In a study by Meinel et al., risk estimation of parameters of PTE on CTPA including increased RV/LV diameter ratio, abnormal septal morphology, reflux of contrast medium into the IVC, thrombus load and central thrombus location was conducted and declared increased RV/LV diameter ratio as the strongest predictive value for adverse clinical outcomes.^31^ Chaosuwannakit et al. evaluated RV dysfunction parameters identified by CTPA, such as the increased RV/LV diameter ratio, interventricular septum deviation, increased RV diameter and reflux of contrast medium into the IVC, and showed significantly higher mortality associated with all of them and the highest adjusted OR for mortality was with RV diameter, followed by PESI score and the CT obstruction index.^32^ Another study by Furlan et al. showed that among the signs of RV dysfunction on CTPA, the RV/LV diameter ratio, main pulmonary artery to ascending aorta diameter ratio, flattening and bowing of interventricular septum, and reflux of contrast medium into the IVC were associated with short‐term mortality and the increase in RV/LV diameter ratio as an only parameter on CTPA independently associated with short‐term mortality.^33^ Nevertheless, reflux of contrast medium into the IVC was recently described as a predictor of mortality in patients with PTE.^34^ This study, as well, aimed to show that among RV dysfunction criteria based on CTPA, PESI score and CHA_2_DS_2_‐VASc score was only correlated with the reflux of contrast medium into the IVC. Shayganfar et al. suggested that the CTPA indices including the presence of reflux of contrast medium into the IVC and the presence of an abnormal interventricular septal morphology as indicators of high PESI score but they did not find any association between RV/LV diameter ratio and PESI score.^35^ This study failed to show a significant association between RV/LV diameter ratio and both PESI score and CHA_2_DS_2_‐VASc score, as well.

Aviram et al. evaluated CTPA parameters among the patients with acute PTE and showed that increased RV/LV diameter ratio was more common and also, only substantial grades of reflux of contrast medium into the IVC was related to 30‐day mortality.^36^ In our study, we detected reflux of contrast medium into the IVC and hepatic veins which means the reflux of contrast medium was significant. Another study showed that reflux of contrast medium into the IVC was associated with PTE‐related and all‐cause mortality^37^, ^38^ and the need for intensive care in PTE patients.^39^ On the other hand, Collomb et al. conducted a cohort on 81 patients and demonstrated no association between reflux of contrast medium into the IVC and severity of PTE.^40^ Also, Lhyne et al. demonstrated that RV/LV diameter ratio >1 and reflux of contrast medium into the IVC did not predict adverse outcomes among patients with acute PTE.^41^

In addition, in this study, we showed that CHA_2_DS_2_‐VASc score and PESI score have a positive correlation with *r* = .523 (*p* < .0001), which was not assessed to date, as far as we are concerned. It is plausible that a number of limitations may have influenced the results obtained. This is a retrospective, observational, and single‐center study with a limited number of patients. Inevitably, a retrospective study design has its own limitations which makes it susceptible to multiple biases. However, we attempted to limit these biases by defining a time frame from which vital signs could be used for scoring. Apart from that, since our data is based on only a single time point, it may not reflect the patient status over long periods. Another possible source of error may be due to the participation of only one radiologist for reading the CTPA and one observer calculating the scores and more observers might have increased the validity of our study. We hope further studies will draw definite conclusions.

## 5 CONCLUSION

This study showed that not only CHA_2_DS_2_‐VASc and PESI scores are positively correlated, but also they are both associated with RV dysfunction diagnosed by CTPA. CHA_2_DS_2_‐VASc and PESI scores are able to predict RV dysfunction and are associated with reflux of contrast medium into the IVC among RV dysfunction criteria on CTPA. Further studies are required to define the role of the CHA_2_DS_2_‐VASc score in acute PTE risk stratification.

## CONFLICT OF INTERESTS

The authors declare no conflict of interest.

## Supporting information

Supporting information.

## Data Availability

The data that support the findings of this study are available on request from the corresponding author.
