# Supplementary material for: CHA2DS2‐VASc and PESI scores are associated with right ventricular dysfunction on computed tomography pulmonary angiography in patients with acute pulmonary thromboembolism
Source: Clin Cardiol. 2022 Feb 7;45(2):224–30. doi: 10.1002/clc.23786 (PMC8860482; doi:10.1002/clc.23786)
Supplement: Supplementary file 1 — Supporting information. [file CLC-45-224-s001.docx]

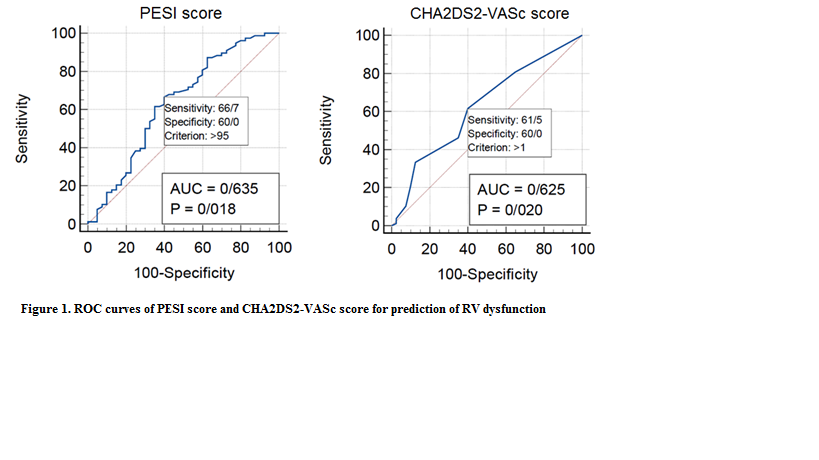


**Supplementary figure 1. ROC curves of PESI score and CHA2DS2-VASc score for prediction of RV dysfunction**
